# Supplementary material for: Construction and validation of an ultraviolet germicidal irradiation system using locally available components
Source: PLoS One. 2021 Jul 23;16(7):e0255123. doi: 10.1371/journal.pone.0255123 (PMC8301618; doi:10.1371/journal.pone.0255123)
Supplement: S1 Appendix — (DOCX) [file pone.0255123.s001.docx]

**S1 Appendix**

**Process implementation**

The evaluation, construction and successful implementation of an N95 decontamination process (by UVGI or other methods) requires participation and involvement by multiple stakeholders throughout the hospital. One significant requirement is that decontamination efforts are set as a priority by facility leadership. Leadership priorities could empower rapid actions toward decontamination, primarily through the commitment of both staff and resources towards the effort, and with an agreement to address any needs as they arise for the effort. Once decontamination has been approved and prioritized from above, the highest priorities would be: 1. Formation of a workgroup to determine modality and feasibility (see below), 2. Assessment of available local resources that could be allocated towards decontamination efforts (for example, UV-C light sources and light meters), 3. Attempts to acquire necessary materials, as these are likely to be amongst the earliest rate-limiting steps, and 4. Determination of institutional regulatory/competency requirements, so that any appropriate forms and SOPs can be approved by required system entities, ideally in an expedited manner. Ideally these could be accomplished by workgroup members in parallel. Finally, used FFR collection should begin as soon as possible, to preserve supply and to provide masks for decontamination once available.

**Workgroup: composition and integration**

We recommend the formation of a workgroup with delegates from front-line healthcare providers, nursing management, sterile processing services, infection control, occupational safety, research staff, facilities staff and electricians, supply chain management, and facility leadership. Each of these groups will play a critical role in either engaging with, or facilitating, decontamination efforts. Possible roles for each group in the effort:

**Healthcare providers:** use and labeling of N95 FFRs, commitment to process

**Nursing management:** implementation, coordination, communication

**Sterile processing:** collection, tracking, regulatory compliance, SOP generation

**Infection control:** decisions re: deployment of decontaminated N95s,

**Occupational safety:** for safety of UVGI technicians, fit testing of FFRs

**Research/Engineering staff**: UVGI system design, technical validation, resources

**Facilities and electrical:** space allocation, facility construction, resources

**Supply chain management:** acquisition of appropriate resources to enable collection

**Facility leadership:** overall management and priority allocation/determination

Each workgroup member should be positively engaged in the process, individually empowered to pursue the best course of action towards efficient and immediate implementation of workgroup decisions, and allowed to prioritize workgroup tasks by direct action without delay. Communication should be carefully managed, to coordinate individual efforts in smaller teams using direct electronic or voice contacts (better for staff that are not typically active at their desks), and intermittent group updates by email to keep efforts coordinated and progress clear. Facility-wide communication should also be coordinated regarding group progress and implementation plans, and ideally mask collection should commence immediately to allow for immediate decontamination at scale once the workflow is implemented.

**Collection and labeling logistics**

At our institution, staff have a strong preference that decontaminated N95 FFRs be returned to the original user, so we developed a procedure for mask collection and tracking based on Nebraska Medicine’s well-established protocol [10]. Additionally, as some pathogens, such as bacterial spores, might survive UV-C irradiation [13], this “return to original user” approach also prevents potential cross-contamination by these agents. At our facility, masks are labeled on the edges with marker, using both a unique identifier based on our local computer login username and the health care worker’s section or department. Alternative labeling techniques using tags or stickers were excluded as they increased the chance of inadequate mask decontamination due to UV light shading. We are not aware of any data indicating that magic marker writing on the edge of the mask could degrade mask material/function, and we did not observe any instances in which magic marker caused masks to fail fit testing (either before or after decontamination).

We recommended that masks be labeled prior to donning, to minimize the potential for provider/workspace contamination after doffing. We also recommended that care be taken with mask straps to avoid damage, as visible strap stretching from even a single use was occasionally significant. Staff were instructed to avoid wearing cosmetics that could build up on mask surfaces and prevent decontamination. Labeling instructions were disseminated to charge nurses at each unit, posted at collection sites, presented repeatedly at staff meetings, and communicated verbally during visits to collection sites, which improved compliance. By the 4^rd^ week after implementation, approximately 80% of N95s were being appropriately labeled which has the potential to substantially sustain PPE availability. We are continuing to work to increase this fraction.

After doffing each N95, users were instructed to carefully place their masks in brown paper bags, to avoid user and bag contamination. Used masks and bags were transiently stored in mask collection bins placed at convenient locations, and providers used a marker at the collection site to label the bag with username, unit, and date. Our sterile processing service collected masks from each site daily, and these were stored in a central location until ready for decontamination.

Our collection and labeling protocol is similar to the Nebraska approach [10], although it differs subtly in the following respects: 1. Labeling does not include the date of first use to improve labeling compliance/ease, 2. Brown bags are folded over at the top and collected in closed carts, and 3. Sterile processing staff have been keeping a log sheet of all returned masks (listed by user/unit). Although tracking of individual masks is not a goal of this effort, it allows for user feedback in terms of mask labeling legibility, and also allows for unit-specific tracking of N95 return rates.

**Workflow**

The decontamination process itself involves a unit-directional flow of masks through our custom-built UV decontamination suite, in which masks are brought in sealed bins to the “dirty” work area, decontaminated in the adjacent (but separated) UVGI room, and then transferred to the “clean” workroom for packaging into white paper bags. These bags are labeled with the provider’s unique identifier, unit, date/time of UVGI cycle. Masks are marked after each cycle on the mask edge. HVAC for each work area was adjusted such that the decontamination room itself was a negative pressure space, while the clean workroom maintained positive pressure relative to the other spaces.

We plan to decontaminate and re-use the N95 FFRs up to 10 times, as long as they continue to pass repeated user-performed seal testing, based on prior studies of multiple decontamination and wear cycles [24]. Our facility predominantly has the Halyard 46728 “Duckbill” masks, and although we have yet to re-issue masks after decontamination, we have had anecdotal reports of inadequate mask seal after several don/doff cycles (without decontamination) due to stretched straps. Although mask straps for this design could potentially be replaced [34], these observations emphasize the importance of seal tests for each decontaminated mask, at each re-use cycle.

**Throughput**

Our current workflow involves hanging masks by their straps on a line between two light sources. This line is connected to hooks on each end, and tensioned by way of rubber bands. The two tensioned lines between the posts can each currently accommodate 20 masks, for the ~30 minute UVGI cycle. Since loading/unloading of the masks occurs within the UV chamber room, it can only be run approximately once per hour. If needed to increase throughput, we will convert to a cantilevered cart system, with protruding arms supporting the tensioned lines. With this improvement, carts could be loaded with masks outside of the UV chamber, while a UV decontamination cycle is taking place. This could increase throughput by about 50-80% without the addition of new UV decontamination lights. Additionally, the addition of reflective material to surround the UVGI chamber could increase the available UV-G energy delivered to each mask, while also improving the number of incident angles, and thus might further increase throughput by reducing exposure times.

**Storage considerations**

Although there are no official guidelines for storage conditions related to post-decontaminated masks, we recommend that masks be stored in a temperature and humidity controlled environment, at least within the manufacturer’s original framework for new masks, but ideally with humidity < 60%. The space required for storage could be substantial, depending on storage modality and the number of masks, and is best estimated with a small group of bagged masks for empiric assessment.

Considerations relating to storage space for N95 FFRs needing decontamination, or decontaminated masks awaiting redeployment, depend greatly on local conditions. Specifically, as new N95s are preferred over decontaminated masks [14], the deployment of decontaminated N95s would likely be coordinated relative to supply/demand balance. However, due to the existence of non-surgical grade FFRs with different performance and fit characteristics, re-deployment of decontaminated, high quality N95 FFRs might be preferable to the use of either non-surgical N95 FFRs or those obtained from community sources.

**Regulatory considerations**

To our knowledge, there are not currently any FDA-approved UVGI N95 decontamination systems. However, the CDC has advised institutions that they could consider decontamination to continue to support their clinical staff [14], and thus, each facility will need to create its own system to provide final approval to the process, likely involving the generation of detailed SOP and Competency documents, similar to those needed for other sterile processing procedures. We would recommend expediting this process, and or obtaining provisional approval to begin decontamination on a provisional basis immediately while paperwork is being finalized, if needed, to prioritize patient/staff protection while regulatory compliance is being addressed.

**References**

10. Lowe JJ, Paladino KD, Farke JD, Boulter K, Cawcutt K, Emodi M, et al. N95 Filtering Facemask Respirator Ultraviolet Germicidal Irradiation (UVGI) Process for Decontamination and Reuse. https://wwwnebraskamedcom/sites/default/files/documents/covid-19/n-95-decon-processpdf. 2020;Accessed online, March 28, 2020.

13. n95Decon.org. Technical Report for UV-C-Based N95 Reuse Risk Management. https://static1squarespacecom/static/5e8126f89327941b9453eeef/t/5e8541760211467623b1e4e4/1585791351199/200401_N95DECON_UV_technicalreport_v12_finalpdf. 2020.

24. Heimbruch B, Harnish D. Research to Mitigate a Shortage of Respiratory Protection Devices During Public Health Emergencies: Appendix 1: Limited Study Evaluating UVGI-Treated FFR Odor. Applied Research Associates, Final Report to the FDA. 2020;HHSF223201400158C(Accessed April 28, 2020).

34. Now T. Team Comes Together to Repair Thousands of N-95 Masks. 2020;(https://now.tufts.edu/articles/team-comes-together-repair-thousands-n-95-masks):Accessed online April 28, 2020.

14. CDC. Decontamination and Reuse of Filtering Facepiece Respirators. https://wwwcdcgov/coronavirus/2019-ncov/hcp/ppe-strategy/decontamination-reuse-respiratorshtml. 2020.
