# Supplementary material for: Construction and validation of an ultraviolet germicidal irradiation system using locally available components
Source: PLoS One. 2021 Jul 23;16(7):e0255123. doi: 10.1371/journal.pone.0255123 (PMC8301618; doi:10.1371/journal.pone.0255123)
Supplement: Attachment — Submitted filename: PONED2015194R2 Response to Reviewers.pdf [file pone.0255123.s003.pdf]

PONE-D-20-15194
Response to reviewers

Overall, although our original reviewers found our manuscript "commendable" and "of interest", the primary concern with this manuscript was the lack of independent verification of decontamination efficacy using live SARS-CoV2 virus samples. As the manuscript's intent was to describe construction and process implementation (not decontamination efficacy), and the data provided validate the physical parameters of the device, the manuscript and title were extensively revised to make this substantially clearer. Perhaps most salient was the change in title to "Construction and Validation of an Ultraviolet Germicidal Irradiation System using Locally Available Components" which we believe more accurately reflects the content of the manuscript, which describes how to construct a UVGI device using commonly available components, and provides additional validation that it delivers a dosage of UV-C light that has already been extensively shown to decontaminate N95 respirator masks.

After our appeal, an additional reviewer (reviewer #3) added comments indicating that the prior comments had been addressed and that the manuscript was technically sound. After further discussion, our senior editor (Dr. Joseph Donlan) acknowledged that although actual SARS-CoV2 testing would not be feasible/necessary, he indicated that the manuscript required "further testing with microorganisms if you wish to retain the conclusion that the device has germicidal properties."

With this resubmission, we not only directly demonstrate that our device kills microorganisms in a dose-dependent manner, but we also develop and describe a novel, low-tech method to create and implement a biological indicator system to test germicidal efficacy. Additionally, in response to Dr. Donlan's suggestion, we have removed the relatively large section of the prior manuscript that provided hospital recommendations for system process implementation, and have extensively revised the manuscript structure to more clearly frame the question and address it using traditional scientific manuscript structure. The systems process recommendations are now provided as an appendix, as it would likely be of value to others who implement a UVGI system, but could be removed entirely if necessary.

Please see our response to the original review, and other minor comments including reviewer #3's comments in the subsequent review, below. Changes to the manuscript are listed in red text.

Major concern:

Reviewer #1: Given the scarcity of availability of N95 masks for the health care workers, the authors effort to develop a procedure, and a UV-C-based used mask decontamination equipment is commendable during the COVID-19 pandemic. The construction and use procedure of this decontaminating unit and workflow are well described, and details are given for others to implement these in their institutions. The authors mentioned correctly: "two critical aspects that must be considered by any N95

mask decontamination program are decontamination efficacy and subsequent respirator performance. An optimal dose of UV-C light must reduce pathogens on the N95 by at least 3-log levels (per recent FDA guidance), without degrading mask function or fit". Although the respirator function is tested after the decontamination procedure, decontamination efficacy is not assessed. The authors defended this issue using theoretical justifications with the UV dose they considered. They should evaluate the decontamination efficacy of their system with some virus inoculation in unused masks.

Reviewer #2: While the topic is of interest there are two major issues in this paper: First, there are no measurements at all of decontamination efficacy, and second the authors do not seem to have taken into account the lesson of their Ref 21, which essentially tell us that because of the different thicknesses of different N95 mask designs, there is more than a 100-fold variation in the required UV doses for the different designs to achieve the same level of decontamination.

Our goal with this manuscript is to provide technical advice on the construction and implementation of a UV-based N95 decontamination system/protocol, and to provide data on the UV-C dosage/irradiance achieved by our system. We do not claim to directly evaluate decontamination efficacy using actual viruses, but instead demonstrate that our unit reliably provides the necessary UV-C dosage. This dosage is already well-established to effectively decontaminate a variety of different N95 respirator models (and as reviewer #2 notes, already more than is necessary for some models). The construction and implementation of the unit using readily available components are well described, and it is exactly this aspect of UV-C based decontamination that is not readily available in the literature, hence the goal of this manuscript.

To address the potential concern that our manuscript might have been mis-interpreted as directly evaluating viral decontamination, we altered the title to remove the specific reference to N95s and more clearly state the objective of the manuscript as follows: "**Construction and Validation of an Ultraviolet Germicidal Irradiation System using Locally Available Components**". Furthermore, in multiple locations within the manuscript, we explicitly clarify that the intent of our manuscript is to describe the construction of our arrays and provide data on the validation of UV-C dose delivered. We also clarify that the choice of dosage is based on previously published data of a range of FFR models, and also that newer FFR models, and newer data, could further refine UV-C dosage requirements, especially for newer FFR models that may only have been developed in the past year. We also explicitly mention highlight some the methodology of Ref 21, which discusses a method for testing UV-C transmittance through FFR materials, and which might be applicable to the use of UV-C light to decontaminate FFRs that have not previously been formally evaluated. Finally, we provide new additional evidence that the system is in fact germicidal, using a surrogate biological indicator assay, which demonstrates that this device provides dose-dependent germicidal activity.

We are not equipped/approved to do direct viral inoculations and subsequent viral infectivity assays, and thus cannot directly demonstrate viral decontamination. To be fair, however: most, if not all, clinically used hospital decontamination systems around the world have not actually been tested to confirm whether their systems kill SARS-CoV2 or any specific pathogens that they are hoping to inactivate. Instead, decontamination systems almost universally use chemical indicators, non-virus-based biological indicators (as above), or electronic measurement devices (as in our case) to monitor quality control and to determine whether they meet the benchmarks needed for each cycle. In an ideal world with intact supply chains, there would be no need to decontaminate N95 FFRs. However given the current situation, it is necessary to use the best available evidence to guide reasonable implementation of local decontamination protocols when needed.

We believe the fact that our manuscript addresses a more limited scope (focusing on construction and validation of UVGI irradiance), as the technical report and data provide are sound, as acknowledged by reviewer #1. We also firmly believe that our description of how to build and validate such a system, using readily available components at many large institutions, to meet (or exceed) validated dosages still remains worthy of publication. This is of particular importance at this point in history, especially as COVID-19 continues to spread to parts of the world with even more constrained resources and supply chains.

Minor concerns:

Figure 2: Single data points are used, and no statistics are provided

The measurements of irradiance were made from fixed locations within the array, and were not expected to change on repeated measurement. However, as bulbs could conceivably vary on a session-to-session basis in terms of their irradiance, we performed repeat measurements of irradiance at specified locations within the array, and revised Figure 2 accordingly to show standard deviations of these repeat measurements. These measurements showed extremely small variance at each location, which is not surprising since the light intensity produced by each bulb was stable over the timecourse of our experiments. However, we note (page 3) that bulb intensity could potentially change over time, which is one reason that we advocate for monitoring of each cycle with a dedicated UV-C luminometer.

Statistics are provided in the text (page 9) for the comparison of the outermost measurement locations with other locations within the array, and the text notes that the outermost regions would produce insufficient UV-C dose given the exposure time utilized, and should not be used for N95 decontamination.

Some typos noted:
Page 9:, …which are could become an issue before degradation from UV-C light

reduces performance….
Page 11: ….in facilities with biomedical research labs and have been also been previously evaluated for N95…..
Page 13: ….by the 4rd week after implementation, approximately 20% of masks still remained incomplete……

We corrected these typos as follows:
Page 9:, …which could become an issue before degradation from UV-C light reduces performance….
Page 11: ….in facilities with biomedical research labs and which have also been previously evaluated for N95…..
Page 13: ….by the 4rd week after implementation, approximately 20% of N95s were still not being appropriately labeled……

Reviewer #3: In the current manuscript, Eric et al. have presented a cheap and efficient way to construct an ultraviolet germicidal N95 irradiation system using locally available components. Though commercial companies manufacture ultraviolet germicidal systems, the global need for such systems is humongous considering the seriousness of the pandemic. I agree with reviewer one that the construction and use procedure of this instrument is described in detail is described in details. The use of the live SARS-CoV-2 virus could have improved the manuscript's quality, but the feasibility of experiments with SARS-CoV-2 is challenging. The theoretical justification can be strengthened using newly published references. The following references are examples (doi: 10.1016/j.ajic.2020.07.022; doi:10.1080/15459624.2015.1018518; doi: 10.1101/2020.10.05.20206953). Moreover, for risk assessment, the authors can use some references for viewers guidelines e.g. ("Technical Report for Heat-Humidity-Based N95 Reuse Risk Management". N95Decon Research Document. Version 1.2, 4/2/2020). The authors have commented on the reviewer's comment. This is an interesting area and can be effective in Covid-19 management. The authors did not mention the scalability of the system. They can mention it. The authors should also include a cautionary note suggesting clearly that this system needs further validation using the SARS-CoV-2 virus for COVID-19-specific use and other microorganisms for use in other scenarios.

We appreciate and have added the additional references. Of note, the first reference (Banerjee et al., 2021, *American Journal of Infection Control*) only used model-based simulations (no apparent technical validation) to evaluate their system design, but they provide excellent theoretical justification.

As suggested, we also note the alternative heat-based inactivation approach and provide references there, as we agree that this is an alternative method that could be used in resource-limited environments. We also provide cautionary advice re: SARS-

CoV2 and other pathogens and specific N95 models (particularly newer models that continue to be developed) in the discussion, and mention scalability in the methods.
